# The Devil Is in the Details! On Regulating Cannabis Use in Canada Based on Public Health Criteria: Comment on "Legalizing and Regulating Marijuana in Canada: Review of Potential Economic, Social, and Health Impacts"

**DOI:** 10.15171/ijhpm.2016.114

**Published:** 2016-08-20

**Authors:** Jürgen Rehm, Jean-François Crépault, Benedikt Fischer

**Affiliations:** ^1^Institute for Mental Health Policy Research, Centre for Addiction and Mental Health (CAMH), Toronto, ON, Canada.; ^2^Addiction Policy, Dalla Lana School of Public Health, University of Toronto, Toronto, ON, Canada.; ^3^Institute of Medical Science, University of Toronto, Faculty of Medicine, Toronto, ON, Canada.; ^4^Department of Psychiatry, University of Toronto, Toronto, ON, Canada.; ^5^Institute of Clinical Psychology and Psychotherapy, Technische Universität Dresden, Dresden, Germany.; ^6^Center of Clinical Epidemiology and Longitudinal Studies (CELOS), Technische Universität Dresden, Dresden, Germany.; ^7^Campbell Family Mental Health Research Institute, CAMH, Toronto, ON, Canada.; ^8^Communications and Partnerships, CAMH, Toronto, ON, Canada.; ^9^Centre for Applied Research in Mental Health and Addiction, Faculty of Health Sciences, Simon Fraser University, Vancouver, BC, Canada.

**Keywords:** Cannabis, Marijuana, Health Burden, Legalization, Regulation, Production, Sale, Medical Marijuana

## Abstract

This commentary to the editorial of Hajizadeh argues that the economic, social and health consequences of legalizing cannabis in Canada will depend in large part on the exact stipulations (mainly from the federal government) and on the implementation, regulation and practice of the legalization act (on provincial and municipal levels). A strict regulatory framework is necessary to minimize the health burden attributable to cannabis use. This includes prominently control of production and sale of the legal cannabis including control of price and content with ban of marketing and advertisement. Regulation of medical marijuana should be part of such a framework as well.

## The Current Situation in Canada: Waiting for a Federal Legalization Framework

Canada will become one of the first countries to fully legalize cannabis consumption on a national level (as announced at the United Nations General Assembly Special Session on drugs 2016),^1^ and Hajizadeh^2^ tries to summarize the potential economic, social and health consequences of such a move. We will argue here that all of these consequences will depend in large part on the exact stipulations (mainly from the federal government) and on the implementation, regulation and practice of the legalization act on provincial and municipal levels (for similar considerations for the United States see).^3,4^ The federal government’s point person on the legalization of marijuana has declared that legalization would be implemented within a public health framework,^5^ and he spoke about strict controls (for a general overview on regulation and public health).^6,7^ However, even with such a framework there are different options, and further, the provinces will likely be given some latitude to regulate legal cannabis. The devil will be in the details.

As a general background, substance policies matter.^8^ Psychoactive substance use is among the leading risk factors for global burden of disease,^9^ and the last decades have shown that wrong policies may even lead to reversals in the monotonous upward trends of life expectancy that characterized most of the last century.^10,11^ The importance for public health of getting substance policies right has been shown in regards to legal substances,^12,13^ illegal substances,^14^ and pharmaceuticals^10^ (for more general discussions see ^8,10,15^).There are also economic costs to society, which depend on the policies implemented,^16^ and these costs affect not only the healthcare system but also the educational, legal, and other systems.^17,18^ The mere fact of whether a substance use is legal or not does not predict the resulting burden of disease, and consequently, legalization may result in negative or positive health outcomes, depending on how the legal and regulatory framework is designed and implemented.

Given the above background and the situation in Canada, the current contribution has two main objectives. First, we will give an overview of how regulations and implementation may impact on the main behavioural drivers of cannabis-related harm. Second, we give a few examples of how the details of these regulations may impact on the actual outcomes.

## Cannabis-Related Health Harms and Policy

As cannabis use* per se* in a legalized framework has no criminal consequences, we need to establish regulations which would reduce the cannabis use behaviours linked to most of the health burden. This current health burden mainly comprises cannabis use disorders as the most important non-fatal health outcome, and injury fatalities, especially traffic injury fatalities^19^ as the most important mortality outcome (for a quantification for Canada see^20,21^). The most important behaviours linked to burden are the following (see also^21^):

- Heavy use/frequent use over time^22,23^
- Mixing of cannabis use and operating machinery (in particular driving a car)^24,25^
- Using cannabis with high tetrahydrocannabinol (THC)^26^
- Smoking cannabis, especially mixed with tobacco^22^
- Using cannabis in early and mid-adolescence^26,27^

How could regulation play a role in reducing these behaviours?

Education and guidelines may play a role in reducing heavy and frequent use (for guidelines see^22^). One way to finance such efforts would be via a dedicated tax, which would be used for prevention, research, education, and treatment. Examples of such taxes exist in the alcohol and tobacco field,^28^ and justification could be derived from classical economic theory.^29,30^ Another way to impact on frequency of use, especially in adolescents (above legal age) and young adults, is via price (and indirectly via taxation). Alcohol and tobacco policies have shown that price is a powerful tool to influence level of use,^31,32^ and specific taxation schemes may even impact on onset of substance use.^33,34^ Finally, again drawing from alcohol and tobacco, a ban on marketing and advertisement contributes to establish cannabis as no ordinary commodity where certain caution in use patterns are required.^15^

*Mixing cannabis use and driving (or operating machinery)* should be avoided independently of the policy environment. Even though there had been studies showing no significant results of cannabis use on driving,^35^ systematic reviews of all relevant studies and subsequent pooling of results show an impact,^24,25^ and the biological pathways on reaction time and psychomotor coordination are similar between operating a car and other machinery.^19,36^ Thus, *per se* laws similar to the ones governing blood alcohol level to prevent such behaviour (ie, no driving or operating machinery with active levels of Δ^9^-tetrahydrocannabinol which could impair reaction time and psychomotor coordination) should be established.^37,38^

*Using cannabis in early and mid-adolescence* poses specific health risks,^26,27^ including risk on the developing brain. Thus, a minimum purchasing age needs to be implemented similar to alcohol^39^ (which has similar or even more detrimental effects^40^).Moreover, this laws needs to be well-enforced, and experience with alcohol has shown that best enforcement can be achieved can be achieved through a state monopoly on sales.^39^

*Using cannabis with high tetrahydrocannabinol content* is becoming more common in some countries,^41^ and the effects on health (compared to lower THC) can be more detrimental.^19,26^ Obviously, THC content can and should be regulated in legalized environments, similar to regulated ingredients in food, alcoholic beverages or other legal substances. This could take the form of pricing policies that make higher-potency products are more expensive than those with lower potency.

*Smoking cannabis*, especially with tobacco, adds additional risk, especially with respect to respiratory disease.^19^ Again, there should be more education on these specific risks, and there should be encouragement of smoke-free and tobacco-free modes of cannabis use in a legalized environment.

Furthermore, there may be some short-term public health consequences of legalization related to cannabis-related emergency department visits,^42,43^ which may be avoided with specific implementations (see recent proposed changes in Colorado as listed in^43^).

## … and Further Details

The above examples show that regulation can contribute to a reduction in behaviours which have been associated with health harm. However, things are not that simple. Much will depend on controlling the way the legal substance is produced and sold (and we will restrict the following discussion to the latter point).

For cannabis another complication comes into play, which does not exist for other legal psychoactive substances like alcohol or tobacco: medical use.^44^ Medical marijuana programs have proliferated in the United States and Canada,^45^ in part because they allowed higher availability of an illegal substance without changing narcotic laws. Depending on the jurisdiction, some of the usual regulatory principles of pharmaceutical product approval are not required, with the consequence that cannabis is frequently prescribed for conditions where its effect is not clear^46^ or may even be detrimental, such as depression or anxiety disorders.^47-49^ In a regulated legal environment, medical use of cannabis should be restricted to disorders where clear evidence of effectiveness has been established through the same rigorous process of approval as other pharmaceuticals, usually via a series of phases ending with randomized controlled trials in humans to establish efficacy in treating certain conditions.^50^ This would ensure avoidance of problems such as mis-indications as mentioned above. It should be stated that medical research with cannabis has historically faced barriers in the United States,^51^ but this is not an issue in Canada.

Even if these principles are adhered to, there is a question of what should happen to currently established cannabis dispensaries in the interim, or in the long run. The controversy in Toronto after the recent police raids of illegal dispensaries provides some illustration.^52^ In these controversies, some argued that no police action should be have been taken because cannabis will be legalized within less than a year, while others maintained that they were justified because the dispensaries violate the current law for medical marijuana. In addition, the type of dispensaries setting up shop in Toronto may not have a place in the new legal framework, but their presence (and increasing numbers) is creating facts on the ground. The longer this persists, the more challenging it will be for the federal government’s preferred legal cannabis framework to succeed.

Again, the situation is not entirely historically new as illegal producers and sellers of alcohol had to be integrated into a new system after the prohibition of alcohol was lifted in North America. This worked quite well, and moonshine and other illicitly produced alcohol currently play little role in either Canada or the United States.^53^ The creation of a state monopoly that offers market prices to producers may be a solution here, which had worked for unrecorded alcohol in Germany at the time.^54^

Thus, while the debate on legalization of cannabis has often been categorical between its proponents and adversaries, the true challenge will be the exact implementation. If Canada does not get these regulations correct, public health problems may be created, with subsequent costs to society, which may exceed the new tax revenues.^16^ On the other hand, if regulations are carefully introduced based on best available evidence (and admittedly some of this evidence will come from other fields),^55^ with independent monitoring and surveillance, and with openness to change in case of negative developments, Canada has a chance to become a leader as an experimenting society.^56-58^

## Ethical issues

Not applicable.

## Competing interests

Authors declare that they have no competing interests.

## Authors’ contributions

JR wrote the drafts for the original and revised version of the commentary. All authors significantly contributed to the text, and have approved of the final version.

## Authors’ affiliations

^1^Institute for Mental Health Policy Research, Centre for Addiction and Mental Health (CAMH), Toronto, ON, Canada. ^2^Addiction Policy, Dalla Lana School of Public Health, University of Toronto, Toronto, ON, Canada. ^3^Institute of Medical Science, University of Toronto, Faculty of Medicine, Toronto, ON, Canada. ^4^Department of Psychiatry, University of Toronto, Toronto, ON, Canada. ^5^Institute of Clinical Psychology and Psychotherapy, Technische Universität Dresden, Dresden, Germany. ^6^Center of Clinical Epidemiology and Longitudinal Studies (CELOS), Technische Universität Dresden, Dresden, Germany. ^7^Campbell Family Mental Health Research Institute, CAMH, Toronto, ON, Canada. ^8^Communications and Partnerships, CAMH, Toronto, ON, Canada. ^9^Centre for Applied Research in Mental Health and Addiction, Faculty of Health Sciences, Simon Fraser University, Vancouver, BC, Canada.
